# Supplementary figures and images for: The role of adaptation in generating monotonic rate codes in auditory cortex
Source: PLoS Comput Biol. 2020 Feb 18;16(2):e1007627. doi: 10.1371/journal.pcbi.1007627 (PMC7048304; doi:10.1371/journal.pcbi.1007627)

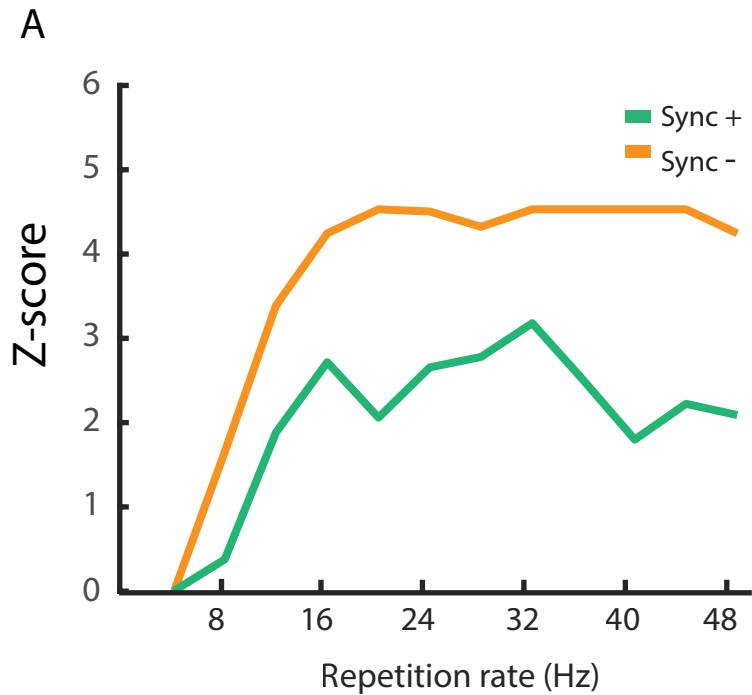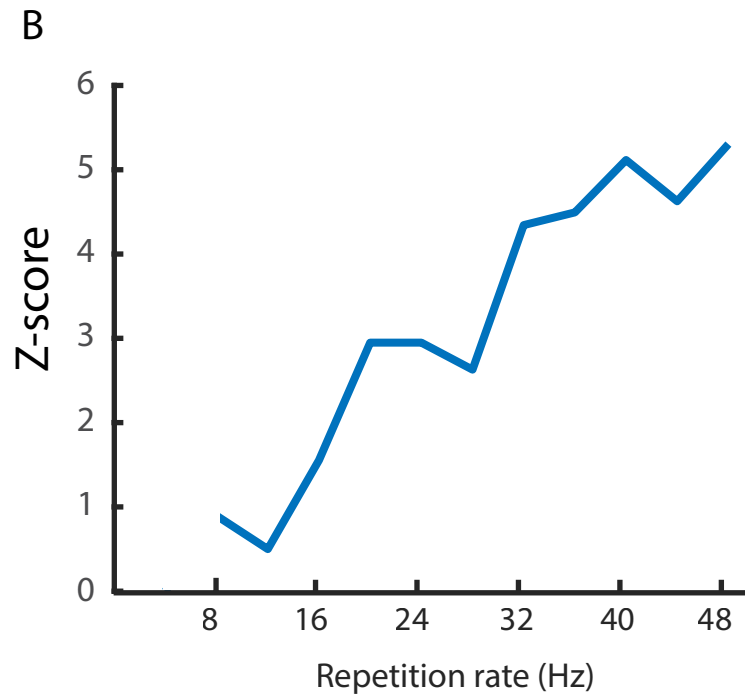

**S1 Fig.** Adaptation to stimuli in Sync+ and Sync- neurons.

Supplement: S1 Fig — For all neurons, we calculated the difference in normalized firing rate between the first and last acoustic pulse for a given stimulus. (a.) For Sync- neurons, this difference was significative for all repetition rates (Wilcoxon signed rank test, P << 0.001) with the exception of 8Hz (Wilcoxon signed rank test, P = 0.10). For Sync + neurons, this difference was significative for repetition rates equal or larger than 16Hz, with the exception of 40Hz (8Hz; P = 0.71. 12Hz; P = 0.06. 16Hz; P = 0.007. 20Hz; P = 0.04. 24Hz; P = 0.009. 28Hz; P = 0.006. 32Hz; P = 0.002. 36Hz; P = 0.01. 40Hz; P = 0.07. 44Hz; P = 0.03. 48Hz; P = 0.04). (b.) We then compared this difference between Sync+ and Sync- neuron populations (n = 25 and n = 26 respectively). This difference was significant for repetition rates above 20 Hz. (Wilcoxon rank-sum test. 8Hz; P = 0.37. 12Hz; P = 0.61. 16Hz; P = 0.12. For higher repetition rates P << 0.01). (PDF) [file pcbi.1007627.s001.pdf]

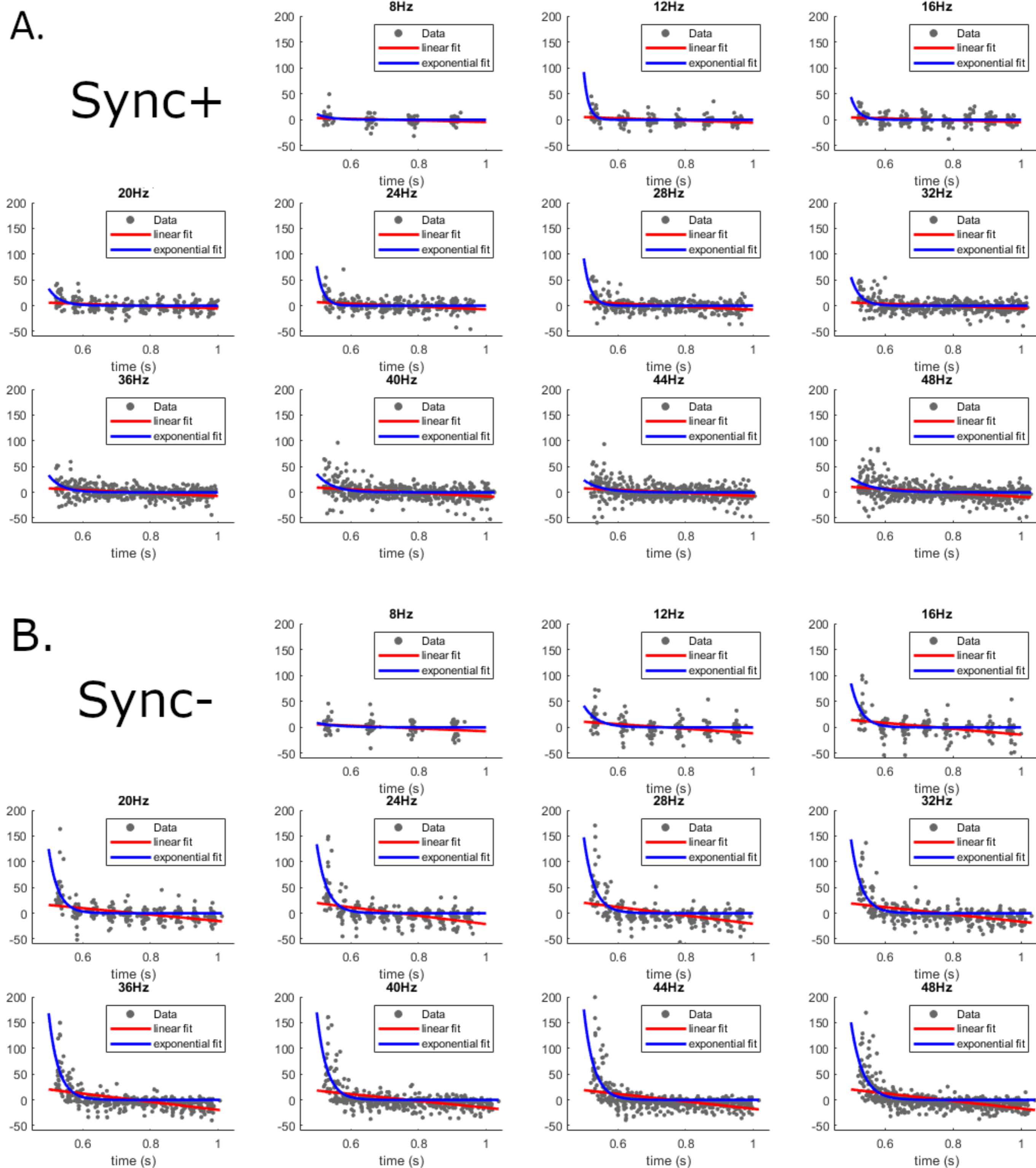

**S2. Fig.** Sync+ (a.) and Sync- (b.) real neuron responses to stimulus pulse trains

Supplement: S2 Fig — Real Sync+ (A.) and Sync- (B.) neuron responses to stimulus pulse trains. For all neurons, the average number of spikes were extracted at each acoustic pulse for all repetition rates. The responses were then normalized by average discharge rate of the neuron during stimulus presentation. Real data (grey), linear fit (red) first degree exponential fit (blue). (PDF) [file pcbi.1007627.s002.pdf]

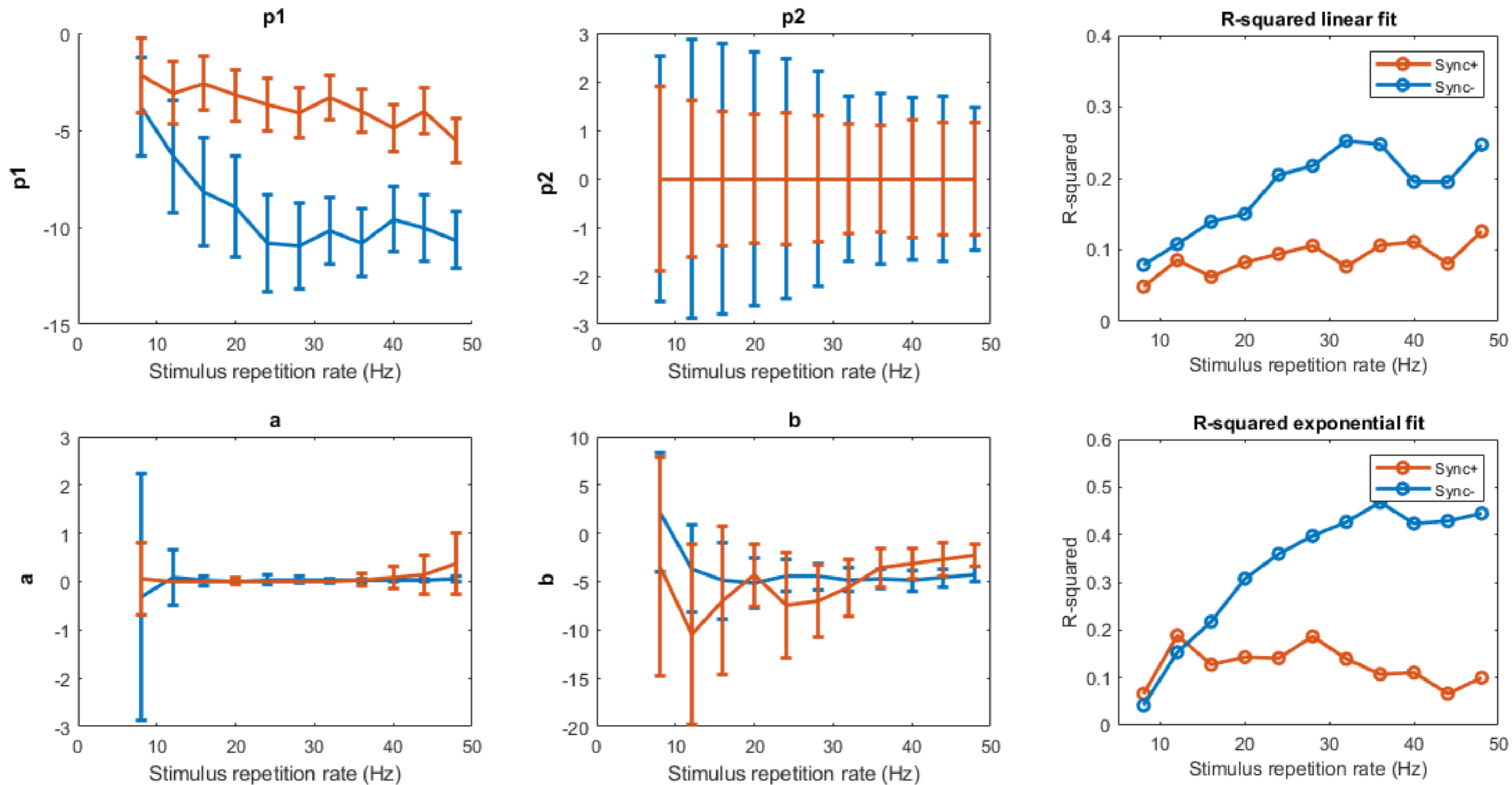

**S3 Fig.** Fitted model coefficients to adaptation during stimulus presentation

Supplement: S3 Fig — (A, B) linear model coefficients with 95% confidence intervals. Stronger negative values of p1 indicate stronger depression during stimulus presentation. (C.) R-squared fit of data to linear model. (C, D) exponential model coefficients with 95% confidence intervals. Stronger negative values of b indicate a steeper curve to the exponential model, indicating a fast adaptation followed by a flat response. Positive values of b indicate no adaptation or facilitation. (PDF) [file pcbi.1007627.s003.pdf]

**D****Onset response  
(spikes/s)**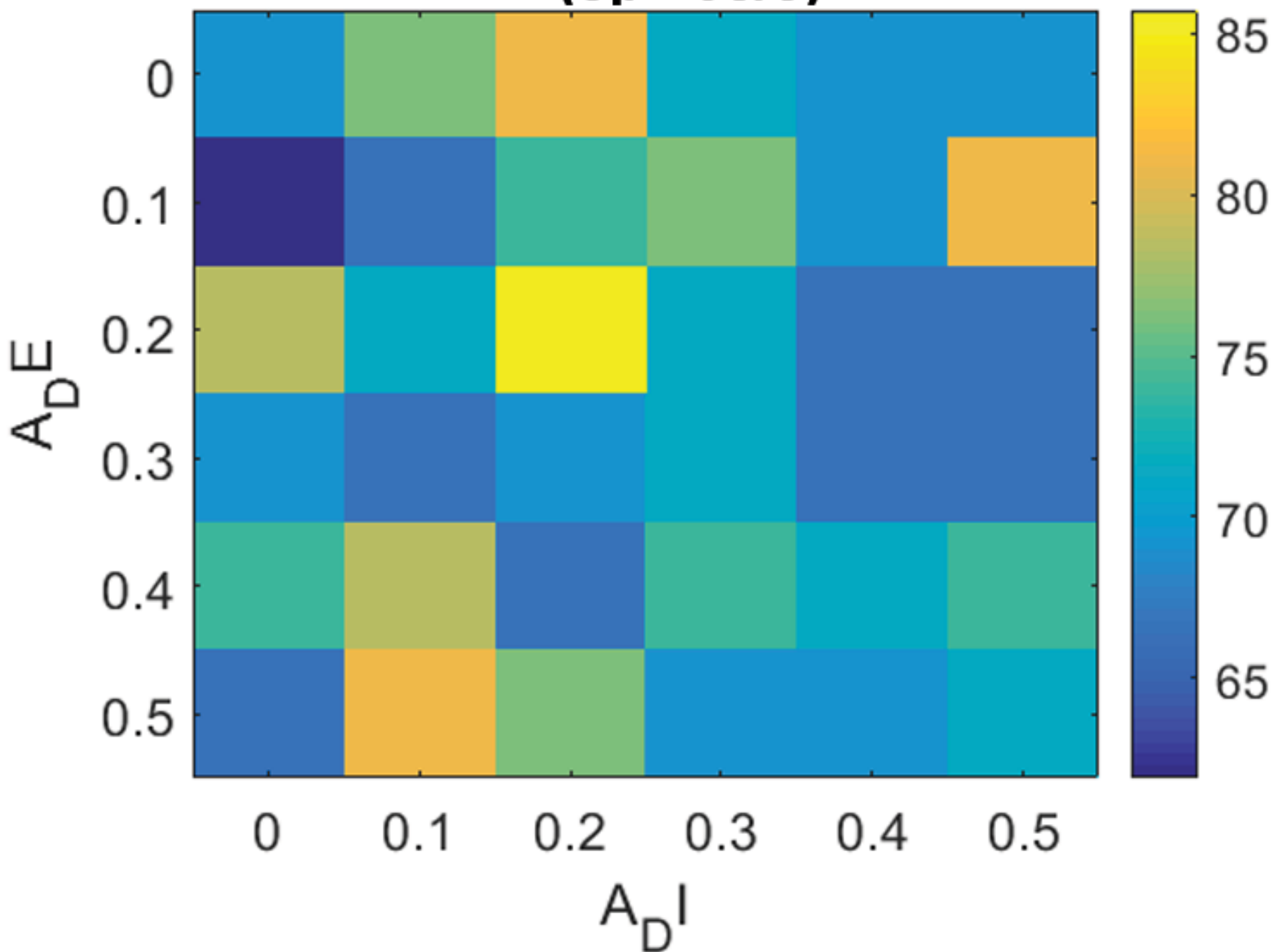

**S4 Fig.** Onset response amplitude relative to strength of adaptation.

Supplement: S4 Fig — Average onset response at time constants {τpE = 0.15, τpI = 0.10} for different values of ADE and ADI. Onset response amplitude did not vary with strength of adaptation. (PDF) [file pcbi.1007627.s004.pdf]

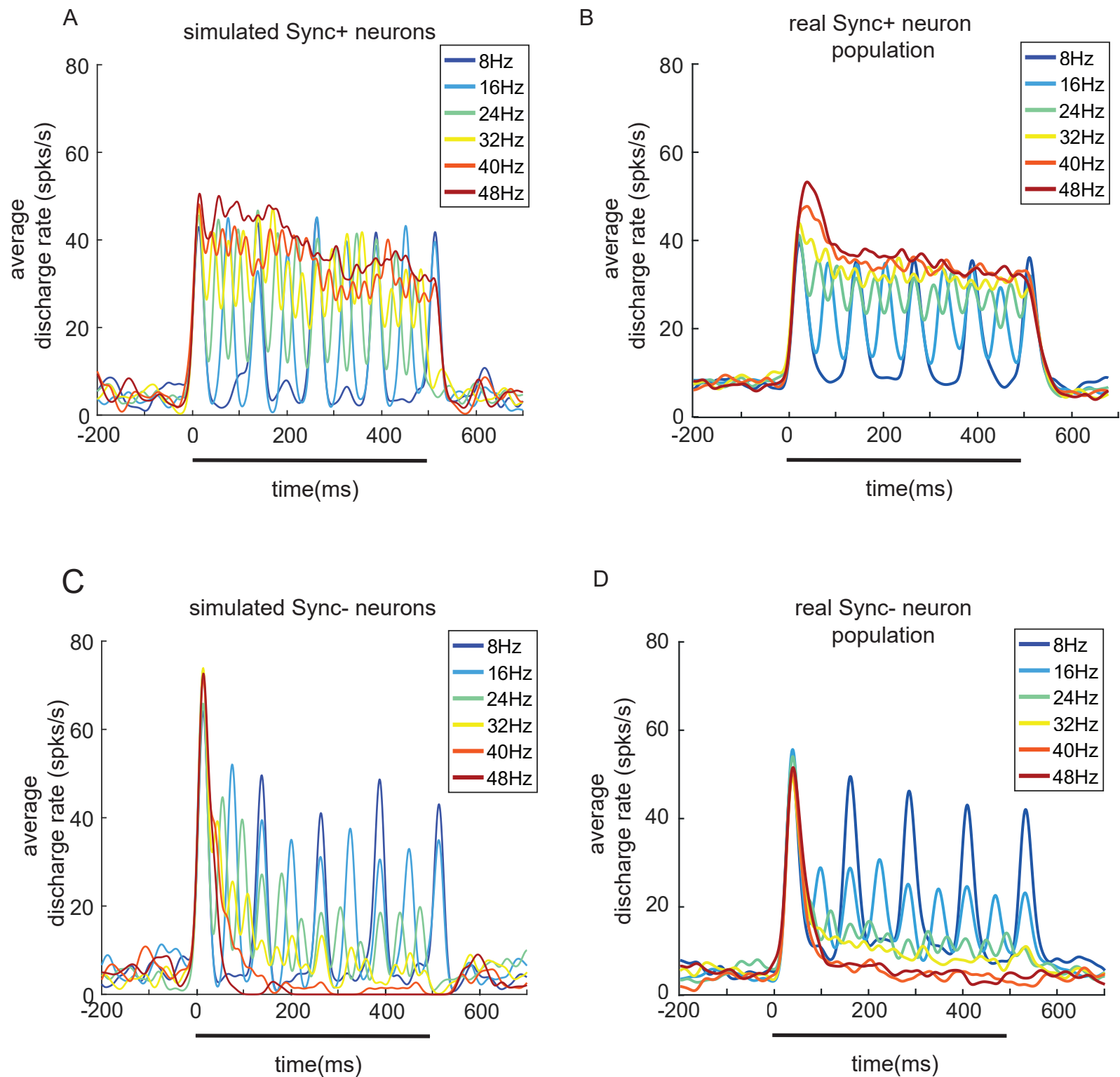

**S5 Fig.** Monotonicity of real and simulated neurons

Supplement: S5 Fig — Comparison between simulated and real neuron population PSTH for Sync+ (A; n = 30, B; n = 25) and Sync- (C; n = 30, D; n = 26) neurons. (PDF) [file pcbi.1007627.s005.pdf]

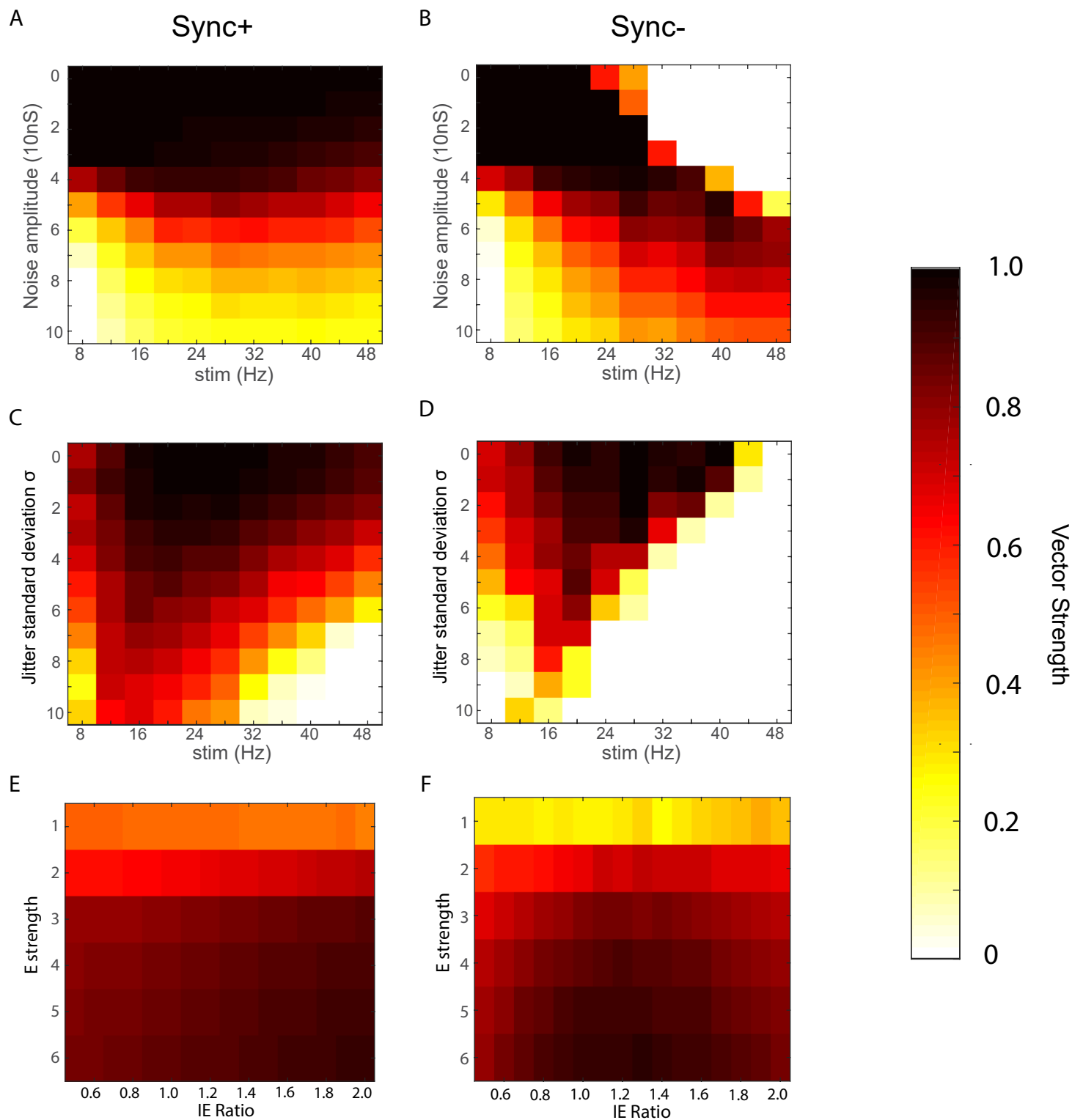

**S6 Fig.** Model robustness regarding vector strength

Supplement: S6 Fig — Vector strength in relation to noise amplitude in Sync+ (A.) and Sync- (B.) neurons, and in relation to temporal jitter in Sync+ (C.) and Sync- (D.) neurons. Average vector strength (E, F.) across different values for recovery time constants {τpE, τpI} ranging between 0.06 and 0.20s for a given value of {ADE, ADI}. Vector strength is maintained for E strength above 2nS and is minimally affected by IE ratio in both scenarios where model parameters produced Sync+ or Sync- neurons. (PDF) [file pcbi.1007627.s006.pdf]

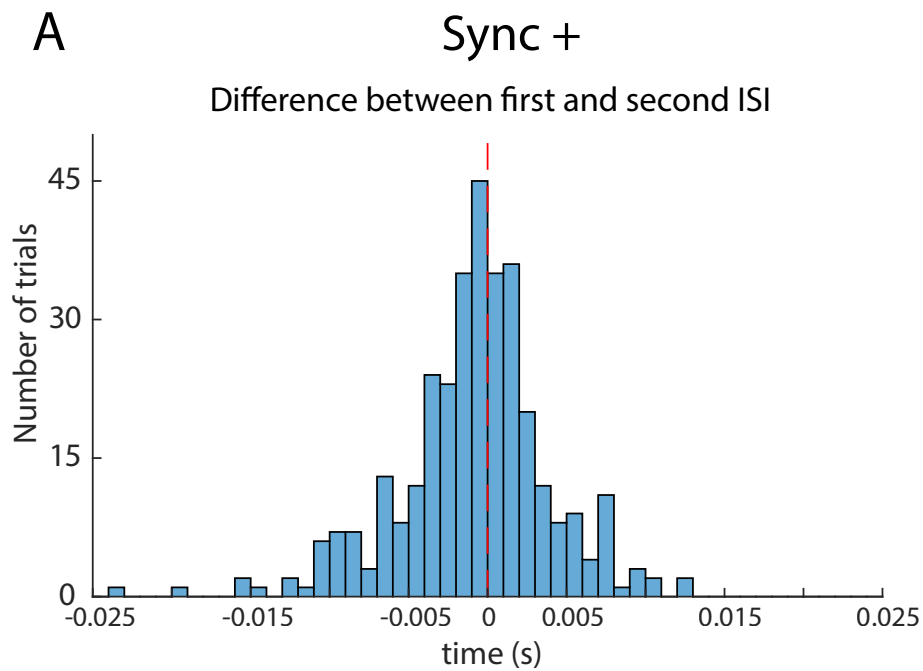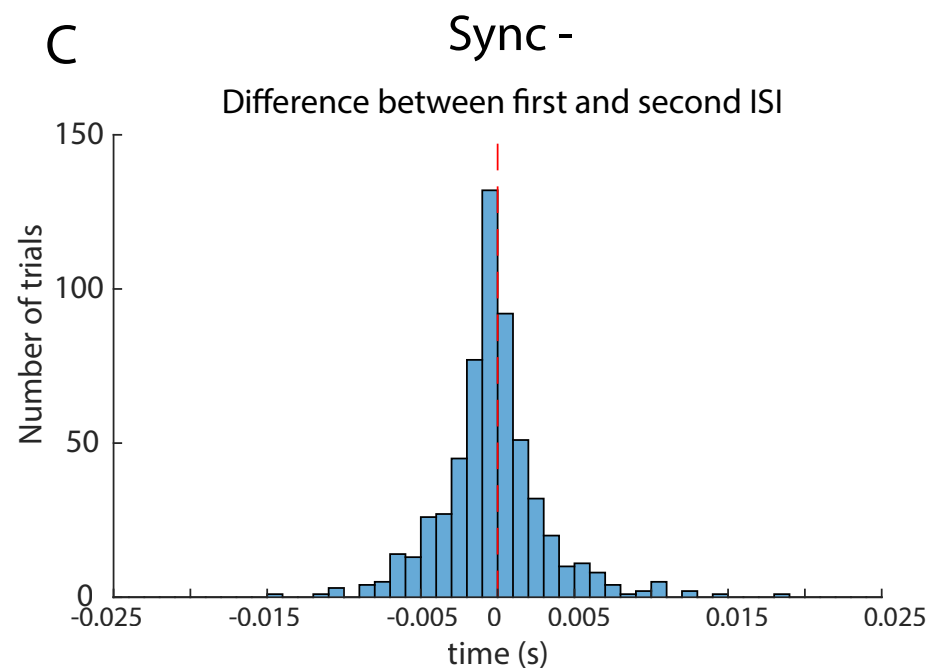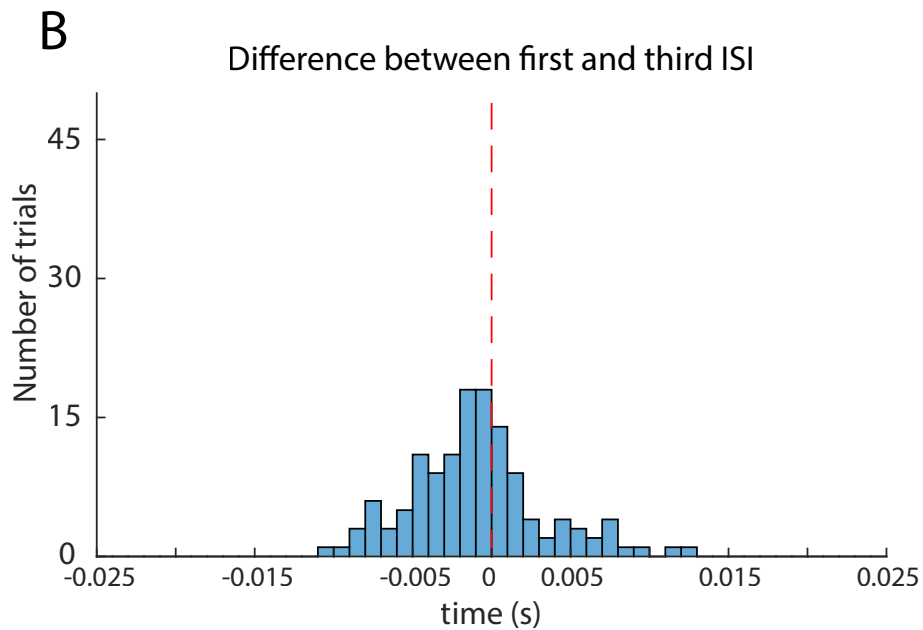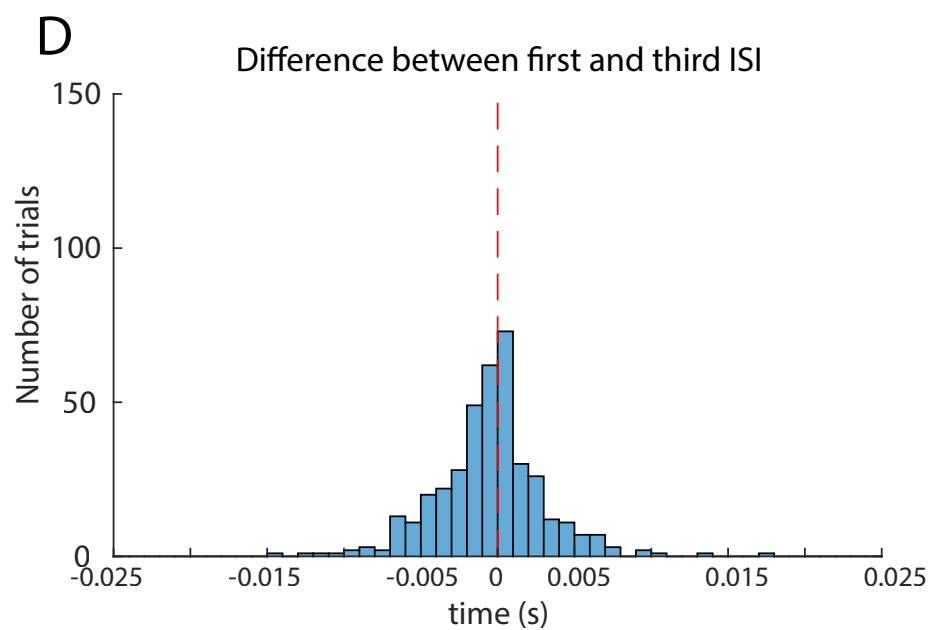

**S7 Fig.** Comparison of ISI after stimulus onset

Supplement: S7 Fig — ISIs between the first four spikes were compared to determine the presence of SRA for real Sync+ (A,B) and Sync- (C,D) real neuron populations for all individual trials across all neurons (n = 250 and n = 260 respectively). All four distributions had a non-zero median (KS test, P < 0.05). For Sync+ neurons, the median difference between first and second ISI was 0.59s (A.) and was 1.21ms for the median difference between first and third ISI (B.). For Sync- neurons, the median difference between first and second ISI was 0.33s (C.) and was 0.24ms for the median difference between first and third ISI (D.). (PDF) [file pcbi.1007627.s007.pdf]

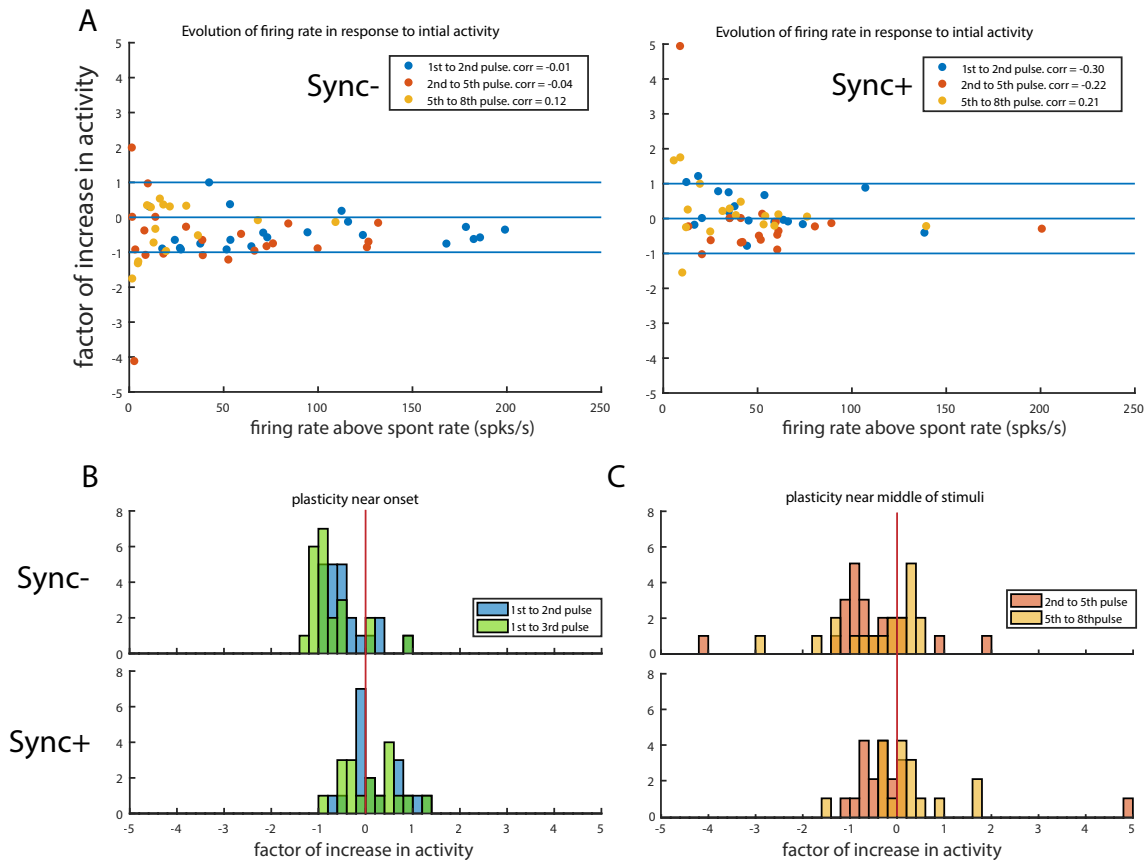

**S8 Fig.** Monotonicity and adaptation in individual neurons

Supplement: S8 Fig — (A). correlation between adaptation and firing rate. Distribution of strength of adaptation near onset (B.) and at the middle of stimuli duration (C.) real Sync- neurons showed significant depression between the first and second (median = 0.73, t-test, P<< 0.001) and between first and third pulse (median = 0.90, P << 0.001) (B.), but not between 2nd and 5th pulse nor between 5th and 8th pulse (median = -0.12, P = 0.33 and median = 0.07, P = 0.51 respectively.) (C.). real Sync + neurons showed no significant depression between 1st and 2nd pulses and between 1st and 3rd pulses respectively (median = 0 for both, t-test, P = 0.12 and P = 0.25 respectively) nor at the later stages of stimuli presentation between 2nd and 5th pulse (median = -0.33, p value = 0.31), and between 5th and 8th pulse, (median = 0.07 p value = 0.54). (PDF) [file pcbi.1007627.s008.pdf]

**A**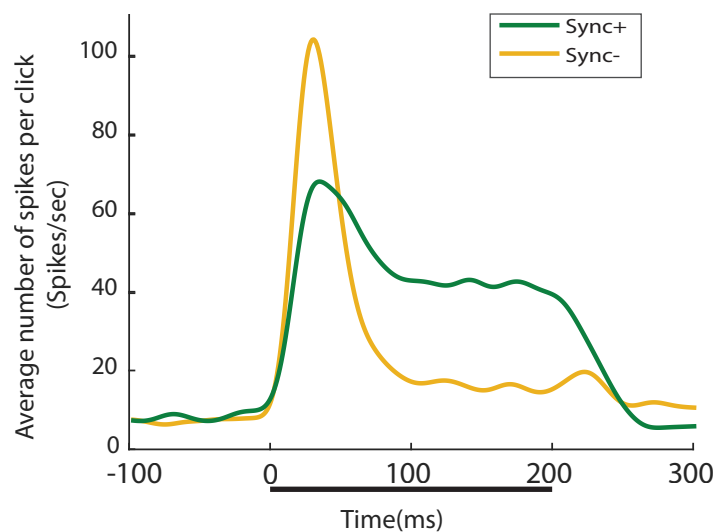**B**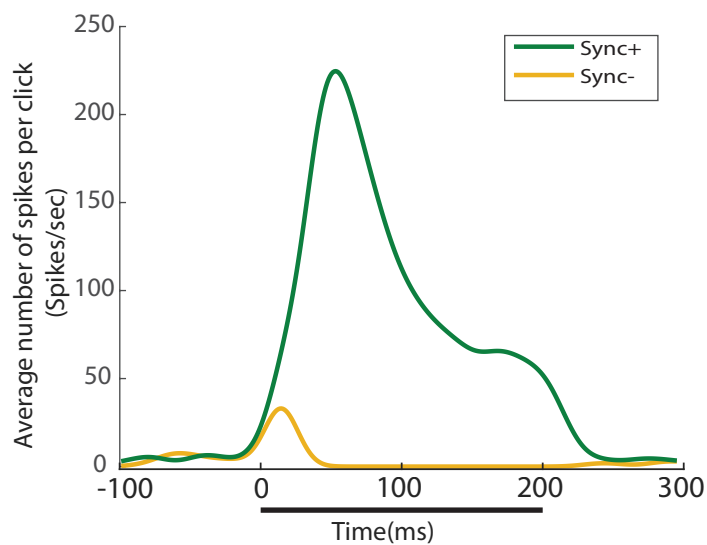**C**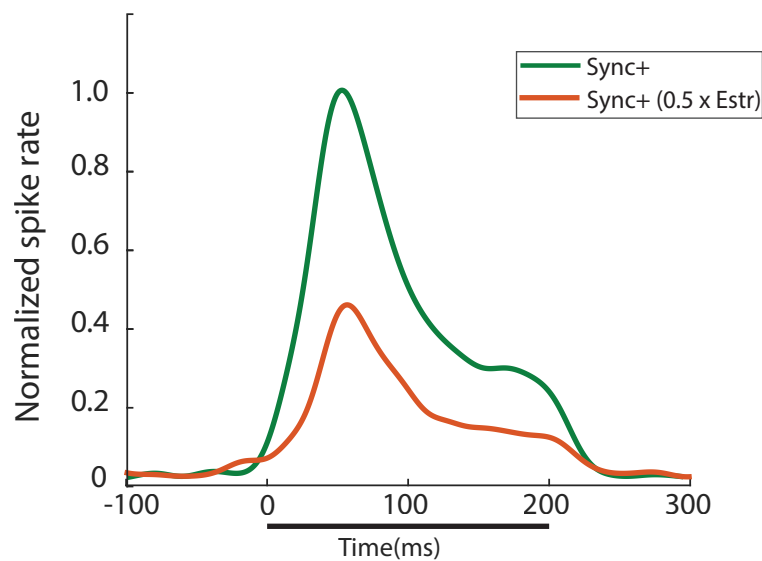**D**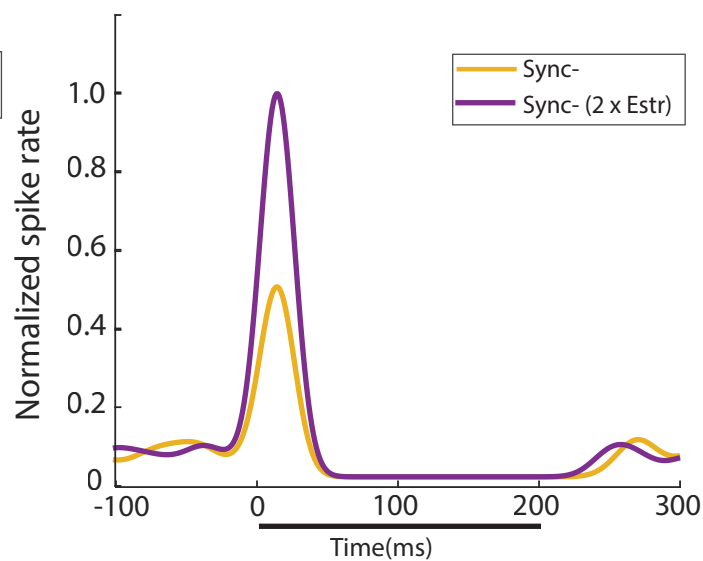**S9 Fig.** Puretone responses

Supplement: S9 Fig — (A.) Average firing rate for simulated Sync+ and Sync- responses to pure tones. (B.) Average firing rate for real Sync + (n = 25) and Sync- (n = 26) neurons to pure tones. (C, D) Effect on varying excitation strength for simulated Sync+ (C.) and Sync- (D.) responses. (PDF) [file pcbi.1007627.s009.pdf]

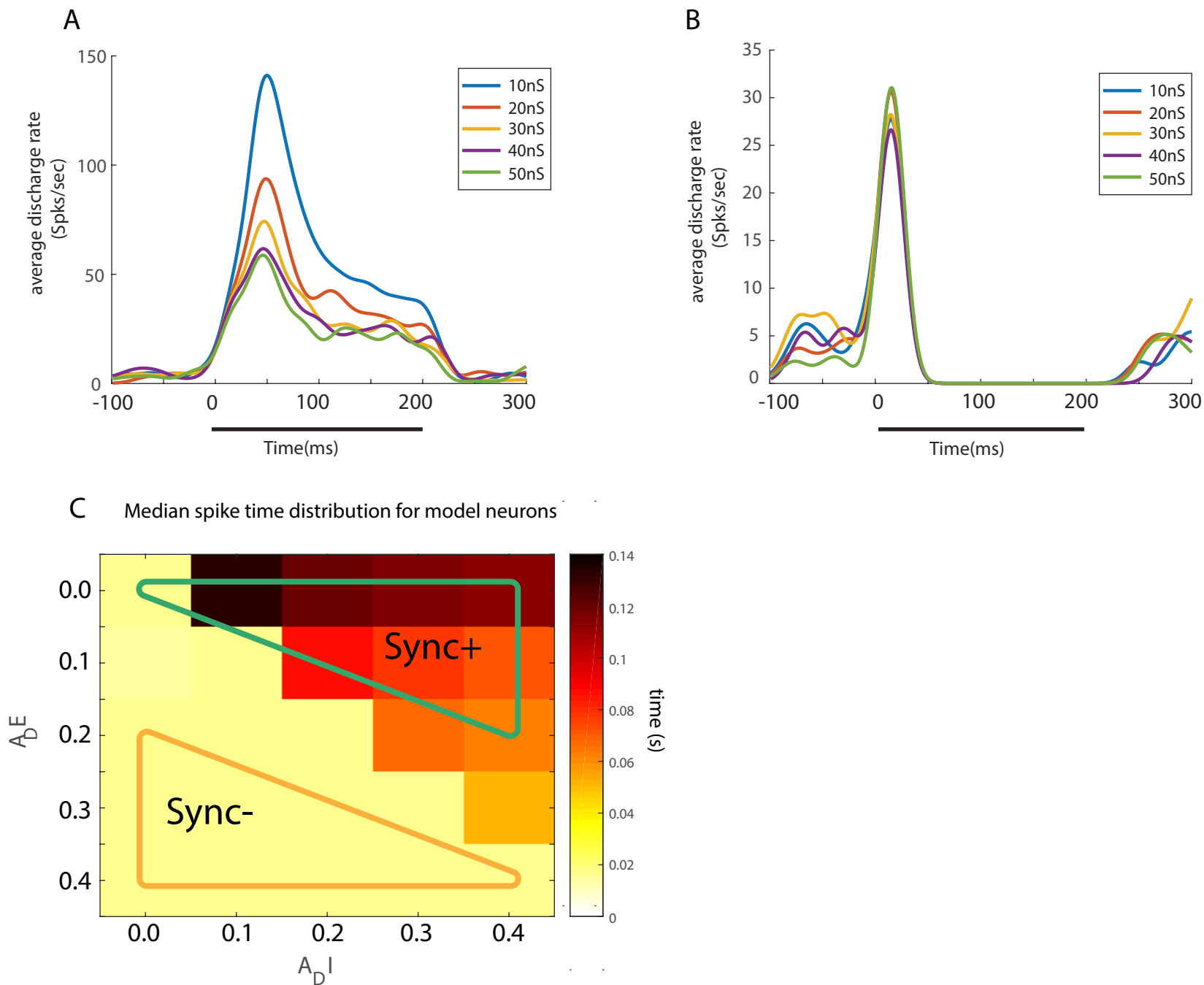

**S10 Fig.** Puretone responses and SFA.

Supplement: S10 Fig — Puretone responses in simulated Sync+ (A.) and Sync- (B.) neurons. SFA was introduced to our model with values ranging between 10 and 50nS (see methods). Stronger SFA reduced both onset and sustained responses on Sync+ model neurons but did not affect Sync- neurons. (C.) Average of median spike times during stimuli presentation for simulated neurons with different values of adaptation amplitude AD. (PDF) [file pcbi.1007627.s010.pdf]
