# Supplementary material for: The role of adaptation in generating monotonic rate codes in auditory cortex
Source: PLoS Comput Biol. 2020 Feb 18;16(2):e1007627. doi: 10.1371/journal.pcbi.1007627 (PMC7048304; doi:10.1371/journal.pcbi.1007627)
Supplement: Attachment — Submitted filename: Review.pdf [file pcbi.1007627.s011.pdf]

What are the main claims of the paper and how significant are they for the discipline?

This study describes a computational model of neurons in the marmoset auditory cortex responding to repeated pure-tone pulses. Both monotonic-positive and monotonic-negative rate responses for SAM modulation frequency or click ISI have been previously described and modelled by the same group (ref. 16), along with neurons that synchronise to slowly repeated stimulus events.

This study additionally highlights a subgroup of neurons that show synchronised responses at low repetition rates, which become primarily onset responses, followed by tonic inhibition. The authors characterise these two populations as sync+ and sync-, since the first group also shows an increase in firing rate as a function of stimulus repetition rate and the second shows a decrease.

Their model very convincingly produces both sync+ and sync- responses exhibited by a subgroup of neurons in the awake marmoset auditory cortex. Furthermore, it is relatively parsimonious and based on realistic physiological mechanisms.

The authors could expand further on the interpretation of these response classes as rate codes for low frequency repeated stimulus events. Why is it useful to consider this a rate-based representation to repetition rate when the previously described sync+ population encode this in their interspike interval? The authors touch on this in the discussion, where they posit that these neurons represent an intermediary stage in the transformation from a temporal representation to a rate-based one, but it would add clarity to expand on this idea.

The authors also mention a recently published study from the same group that proposed a computational model of these responses (ref. 16). This study did not note the presence of sync-neurons and thus did not present a model to explain this. Can the model present in this study also produce non-synchronised responses at high stimulation frequencies? i.e., can it reproduce sync+, sync-, nSync+ and nSync- responses, using physiologically plausible parameter relationships? The Discussion could be improved by contrasting the two approaches in more detail.

Are the claims properly placed in the context of the previous literature? Have the authors treated the literature fairly?

The authors could mention some of the literature on why a transformation to rate code is generally thought to be required (i.e., biophysical limitations of cortico-cortical transmission).

Do the data and analyses fully support the claims? If not, what other evidence is required?

Yes

Are original data deposited in appropriate repositories and accession/version numbers provided for genes, proteins, mutants, diseases, etc.?

The authors have provided a Github repository, which presumably contains the code to reproduce the data from their simulations. They could consider creating a DOI linked to the version of the code that was used in the study for posterity (https://guides.github.com/activities/citable-code/). The authors have indicated that all data are fully available, but I cannot see a link to the simulation and electrophysiological datasets.

Are details of the methodology sufficient to allow the experiments to be reproduced?

The model is developed such that it reproduced spiking responses observed in neurons in a previously published dataset. The authors report a dataset comprising 210 neurons, primarily from the auditory cortex. However, in the reference given describing this dataset this number is higher (274). It's not clear how this subset was selected.

The duration of the pulses is described as "brief", but it would be nice to quantify that.

It seems likely that the stimulus intensity could have a profound effect on how these neurons behave. It is indicated that the stimuli were played 10-30 dB above threshold, but did the threshold differ between the sync+ and sync- populations?

Line 363 mentions that stimuli were presented at a different level for neurons with non-monotonic rate functions, but this group of neurons is not discussed in the paper.

The following sentence (line 361) appears to be directly taken from ref. 15.

Is the manuscript well organized and written clearly enough to be accessible to non-specialists?

The manuscript is well organised and written clearly.

Typos

Line 345: "…data in this report comprised of previous…" should be "data in this report comprised previous".

Line 173: "…as opposed to depression of adaptation." should be "…as opposed to depression of excitation."
