# Supplementary material for: The role of adaptation in generating monotonic rate codes in auditory cortex
Source: PLoS Comput Biol. 2020 Feb 18;16(2):e1007627. doi: 10.1371/journal.pcbi.1007627 (PMC7048304; doi:10.1371/journal.pcbi.1007627)
Supplement: Attachment — Submitted filename: response to reviewers_v6.pdf [file pcbi.1007627.s012.pdf]

We thank the reviewers for their constructive and helpful remarks and greatly appreciate the efforts they made in providing detailed comments on our manuscript. The following is a point-by-point list of all changes made in response to the comments by the three referees. All page and figure numbers referred to here are those of the revised manuscript.

Reviewer #1

The authors could expand further on the interpretation of these response classes as rate codes for low frequency repeated stimulus events. Why is it useful to consider this a rate-based representation to repetition rate when the previously described sync+ population encode this in their interspike interval? The authors touch on this in the discussion, where they posit that these neurons represent an intermediary stage in the transformation from a temporal representation to a rate-based one, but it would add clarity to expand on this idea.

Rate-based representation to repetition rate represent processed temporal information, as opposed to preserved temporal information in ISI based representations. With rate coding, cortical processing of auditory streams would operate on a “segment-by-segment” basis, and not on a “moment-by-moment basis” as found in the auditory periphery (Wang et al 2008). As previous studies (Bendor and Wang 2007, Wang et al 2015) have suggested, this would allow temporal integration of information over specific time intervals, which are required by higher-level processing tasks. The transformation from temporal to rate code would be necessary for such complex cortical integration.
Rate coding of temporal information such as repetition rate is thought to emerge in A1/MGB, and only in auditory cortex we observe both temporal and rate coding for the same repetition rate. For these two reasons, we posit that the Sync+ and Sync- neurons studied in this manuscript represent an intermediary stage in the transformation from temporal to rate coding.
We have included these paragraphs in the revised manuscript on page 6.

The authors also mention a recently published study from the same group that proposed a computational model of these responses (ref. 16). This study did not note the presence of sync neurons and thus did not present a model to explain this. Can the model present in this study also produce non-synchronised responses at high stimulation frequencies? i.e., can it reproduce sync+, sync-, nSync+ and nSync- responses, using physiologically plausible parameter relationships? The Discussion could be improved by contrasting the two approaches in more detail.

We agree with the reviewer of the importance to compare the two models in detail. We have expanded the discussion on page 6 as follows:
A previous study (Gao et al 2016) proposed a similar model incorporating short-term depression to produce Sync+ and Sync- responses. Despite the similarities in the approach, the model described in this study differs from the current model in two major ways. First, the previous model deviated from a widely-used STD model (Tsodyks et al) by reducing the probability of release (see methods) to 0 each time a spike is occurred. Whereas the STD model by Tsodyks and colleagues has been fitted to and compared with real data (6,22,23-27), there are currently no previous studies supporting the more simplified model. Second, we define Sync+ and Sync- neurons as neurons having monotonic positive or

negative, synchronised responses within the perceptual range of flutter, as seen in real neurons (Bendor and Wang 2007). The previous model calculates monotonicity across the perceptual ranges of both flutter and fusion, but the resulting Sync- and nSync- simulated neurons do not seem to be monotonically negative within the range of flutter. Therefore, this model is not accurately modelling real neurons observed in Bendor and Wang 2007. We decided to improve upon these two areas to make the model more realistic and more accurately emulate Sync+ and Sync- neurons observed in previous data. Additionally, we do not vary the temporal integration window of neurons (a key model parameter in Gao et al. 2016), as there is a limited experimental data defining the biologically realistic range in auditory cortex.

The authors could mention some of the literature on why a transformation to rate code is generally thought to be required (i.e., biophysical limitations of cortico-cortical transmission).
Previous studies have shown a lower synchronization boundary to Inter-click intervals in MGB (median synchronization boundary: 5.2ms) compared to in A1 (median synchronization boundary: 21.3ms Bendor and Wang 2008). In addition, the loss of temporal fidelity leads to a loss of information unless a non-temporal (rate) representation is used to encode this same information.

The authors have provided a Github repository, which presumably contains the code to reproduce the data from their simulations. They could consider creating a DOI linked to the version of the code that was used in the study for posterity (https://guides.github.com/activities/citable-code/). The authors have indicated that all data are fully available, but I cannot see a link to the simulation and electrophysiological datasets.
The electrophysiological dataset will be made available upon reasonable request to the authors. The revised manuscript has been corrected to indicate this.
The code used to produce simulations are provided in the Github repository in the following link: https://github.com/dbendor/EI-model

The model is developed such that it reproduced spiking responses observed in neurons in a previously published dataset. The authors report a dataset comprising 210 neurons, primarily from the auditory cortex. However, in the reference given describing this dataset this number is higher (274). It's not clear how this subset was selected.

The dataset in the previous paper (210 neurons) only included neurons tested with repetition rates spanning both fusion and flutter perception. In this manuscript (and Bendor and Wang 2007), we include neurons tested with repetition rates spanning flutter perception. As some neurons were tested only with repetition rates in the flutter range (but not fusion), this has led to a high number of neurons overall.

The duration of the pulses is described as "brief", but it would be nice to quantify that.
We have added this modification in the revised manuscript with the following text in page 9 of the revised manuscript:
Pulse widths ranged from σ = 0.89 to 4.65ms. Repetition rates ranged from 4Hz to 48Hz (in 4Hz steps) . The pulse train stimuli were 500ms in length, with at least a 500ms pre-stimulus period and a 500ms post-stimulus period. The number of repetitions for each stimulus was at least five, and at least ten for 55% of neurons (236/274). Stimuli were presented in a random order, and intensity levels were determined based on sound level tuning of individual neurons: generally 10 – 30dB above BF pure tone

thresholds for neurons with monotonic rate-level functions, and at the preferred sound level for neurons with non-monotonic rate-level functions. This was done so that sound levels were above threshold, but not high enough to cause a significant widening of frequency tuning (monotonic RL) or lead to a suppression of the neuron's response (non-monotonic RL).

It seems likely that the stimulus intensity could have a profound effect on how these neurons behave. It is indicated that the stimuli were played 10-30 dB above threshold, but did the threshold differ between the sync+ and sync- populations?
The threshold did not differ between the two populations of neurons (Wilcoxon rank sum test, P = 0.09). There is some confusion between monotonic rate level and monotonic tuning to repetition rate (these are independent of each other). We have clarified this in the Methods section.

Line 363 mentions that stimuli were presented at a different level for neurons with non-monotonic rate functions, but this group of neurons is not discussed in the paper.
This section refers to the sound level tuning curve (monotonic or non-monotonic). Our data set includes both types of neurons, and this does not impact the tuning for repetition rate. The sound level used is above threshold, but not too loud such that it results in a widening of the frequency tuning (monotonic RL) or leads to suppression of the neuron (non-monotonic RL).

The following sentence (line 361) appears to be directly taken from ref. 15.
see response to a previous comment.

Line 345: "…data in this report comprised of previous…" should be "data in this report comprised previous".
Line 173: "…as opposed to depression of adaptation." should be "…as opposed to depression of excitation."

We have made these corrections in the revised manuscript

Reviewer #2
MAJOR CONCERNS.

1. (LL. 168-192) "Different mechanisms…" The manuscript explores two possible alternative mechanisms for producing rate coding, which is helpful, but it seems somewhat limited if the authors really want to claim, as they do in the abstract, that STD "best explains" the rate coding effects. For example, what about inhibitory feedback? Or slow Ih currents? Where exactly in the circuit are the authors postulating that the STD takes place? Testing a broader set of models would be great. But at the very least, the authors should provide a more comprehensive review of possible mechanisms, particularly at the network level. The authors might consider, for example, work by the Geffen lab related to stimulus specific adaptation (Natan et al. 2015). It is also not clear how the current model difference from Gao et al. 2016.

We agree with the reviewer on this important point. There are many types of adaptation in the auditory system (malmierca, Perez Gonzalez 2014), including Stimulus Specific Adaptation (SSA) which has been widely studied in the auditory cortex in mammals. However, the timescale of SSA in previous studies range from 0.25Hz to 8Hz, with 8Hz being considered a fast Inter Stimulus Interval (Nelken 2003, Nelken 2011, Malmierca 2009, Zhao). The stimuli we use in our study are gaussian click trains with repetition rates ranging from 8Hz to 48Hz. If the adaptation was due to some form of SSA by, for example, Slow Ih currents, we should be able to observe a longer timescale for adaptation, even after stimulus presentation. To examine this, we have compared the spontaneous rate 100ms before stimulus presentation to the spontaneous rate 100ms after the stimulus. Our results did not show any evidence of a difference between the two spontaneous rates for both low (8 to 32Hz) and high (36 to 48Hz) repetition rates (Wilcoxon rank-sum test, P = 0.10 and P = 0.43 respectively)

As for top-down inhibitory feedback, we believe that this is unlikely because of the timescale of such adaptations. Adaptation in neurons studied in this manuscript occurs during the second pulse of the click-train stimulus. (supp figure 6), on a timescale of milliseconds. However, for top-down inhibition to occur in response to stimulus repetition rate, at least two pulses of the stimulus needs to be integrated downstream, then feedback to auditory cortex to modulate the driven response to the stimulus, which would likely occur on the timescale of at least tens of milliseconds. Therefore, we believe that adaptation observed in this manuscript is unlikely to be due to top-down inhibition.

We thank the reviewers for underlining the importance of presenting and comparing different potential mechanisms for adaptation in the auditory cortex, and have included the following text in the discussion section in the revised manuscript in page 6:

*Another possible mechanism for adaptation to repeated stimuli is stimulus specific adaptation (SSA) which has been widely studied in the auditory cortex in mammals [*Malmierca et al, 2009*, Duque et al 2012, Duque et al 2014, Malmierca et al 2014). Although we did not model the effects of SSA in this study, previous groups have shown that the timescale for SSA (0.25Hz to 8Hz) to repetitive stimuli is much slower than the perceptual range of flutter (8-45 Hz) [Nelken 2003, Nelken 2011, Malmierca 2009]. This led us to believe that Sync+ and Sync- responses found in auditory cortex were not a result of SSA.*

And in page 7:

Second, our model does not account for different sources of inhibition top-down inhibitory feedback or cortical inhibition from different interneurons that could affect excitatory pyramidal cells in the auditory cortex. There has been increasing evidence of top-down signals mediating rapid, behaviourally driven modulatory effects in the auditory cortex [Mesgarani and Chang 2013, Fritz et al 2003, Atiani et al, Bagur et al]. A recent study has shown that a network of Parvalbumin-positive (PV) inhibitory neurons were able to rapidly and efficiently modify sensory responses in auditory cortex in mice, and may mediate such top-down control [Harrison et al]. Adaptation in Sync+ and Sync- neurons occurred within the order of milliseconds, and the spontaneous rate of cortical neurons did not decrease for stronger adaptation as it did with

activation of PV inhibitory neurons. However, we cannot rule out possible effects of inhibition from other interneurons with shorter, more transient timescales as observed in somatostasin (SOMs) and Vasoactive Intestinal Polypeptide (VIPs) expressing interneurons[Blackwell and Geffen 2017, Wood et al 2017]. Further studies using optogenetics to manipulate different types of inhibitory neurons may provide additional insight on how inhibition affects adaptation in auditory cortex, and the respective roles of feedforward and feedback connections in monotonic tuning.

2. (LL. 241-267). The section on multiplexing is interesting, but does it have anything to do with the initial question of mechanism producing non-phase locked responses? It seems out of place and somewhat trivial in how it is tested. Could it not work? It also seems related to previous work on color opponency, which is not mentioned. Currently an outsized portion of the Discussion focuses on this topic, which is strange, given the nominal focus of the manuscript. It would be help if there were a clearer connection to the rest of the study or perhaps if the study were introduced differently.
In response to comments of multiple reviewers, we've decided to remove this section, and will focus a separate manuscript on the concept of multiplexing.

LESSER CONCERNS

L. 55. The authors might want to specifically mention the somatosensory system by name here, since quite a few of the relevant concepts were developed there.
We agree with the reviewers and have added this to our revised manuscript in page 2.

L. 67. "repetition rate ranged beyond acoustic flutter" It is not clear what exactly the problem is here. Can the authors clarify what is different about this model vs. the current STD model and what it fails to account for?
There are two important caveats to the previous model. First, the previous model deviated from the well-known STD model (Tsodyks et al) by reducing the probability of release (see methods) to 0 each time a spike is occurred. Whereas the STD model by Tsodyks and colleagues has been fitted to and compared with real data (6,22,23-27), there are currently no previous studies supporting the more simplified model. Second, we define Sync+ and Sync- neurons as neurons having monotonic positive or negative, synchronised responses within the range of flutter, as seen in real neurons (Bendor and Wang 2007). The previous model calculates monotonicity across both flutter and fusion, but the resulting Sync- and nSync- simulated neurons do not seem to be monotonically negative within the range of flutter. Therefore, this model is not accurately modelling real neurons seen in Bendor and Wang 2007. We decided to improve upon these two areas to make the model more realistic and more accurately emulate neurons observed in previous data.
A paragraph in the discussions section (page 6-7) has been dedicated to compare the two models as explained above.

L. 71. The authors may be interested in a recent preprint from the David lab (Lopez Espejo et

al 2019) showing a trend toward stronger synaptic depression in excitatory vs. inhibitory inputs in auditory cortex during natural sound processing. Is this relevant to the predominant adaptation of excitatory inputs required for their model.

The trend towards stronger synaptic depression in excitatory inputs observed in the said preprint is in line with the importance of such adaptation for our model. We have included the following text in the revised manuscript in page 7:

In addition, our findings suggest that synaptic depression of excitation may play a role in diversifying how the auditory cortex encodes stimuli. This observation was in line with a recent study [33] which demonstrated an important role of synaptic depression of excitation during natural sound processing in the ferret auditory cortex.

LL. 86-92. Something is not clear in the logic of these sentences. Does the I/E model fail to produce significant sync- effects, even with biologically implausible parameters? Or only if the parameters are forced to be realistic?

The previous I/E model can technically produce higher firing rates at lower repetition rates only when using biologically implausible parameters (I/E ratios much greater than typically observed, Wehr and Zador 2003).However these responses are unlike typical Sync- neurons as they exhibit weak responses at low repetition rates, and completely suppressed responses at middle to high repetition rates. Real Sync- responses generally have well-driven responses (above the spontaneous rate) over the entire range of flutter perception. Thus Sync- neurons cannot be the by-product of extremely strong inhibitory inputs.

L. 118. Please cite the source for the STD model in the results. Currently it's only mentioned in the Methods.

L. 126. "Naturalistic range" Please provide a citation.

We have corrected the manuscript to include these two comments. The citations are included in the "Model parameters" section in page 3.

L. 129. The monotonicity index is introduced with no definition, and it is not clear exactly how this relates to the "sync+" and "sync-" categories even after reading through the methods. In particular, how is "monotonicity" related to synchronous vs. asynchronous responses? It would help if earlier in the results there was more explication of how rate/temporal coding is analysed before getting into the details of the various models.

L45 to 47 defines Sync+ and Sync- categories. In the methods, L427 and L447 describes how we classify Synchrony (temporal coding) and monotonicity (rate coding), both of which are defined independently. When a neuron was classified both as Sync and monotonic positive/negative, it was classified as Sync +/-.

L. 131. Please provide units for tau parameters.

We have corrected the manuscript in page 3 to include these comments.

LL. 157-58. This observation suggests that Sync- neurons might show differences in their phase-locking relative to Sync+ neurons. Is this the case?

Across all repetition rates, VS between Sync+ and Sync- neurons were not significantly different (Wilcoxon rank sum test, P = 0.55). However, for high repetition rates (36 to 48Hz) Sync+ neurons and Sync- neurons showed differences in phase-locking (Wilcoxon rank sum test, P = 0.005), however this is at least partially due to driven rates be significantly less in Sync- neurons at higher repetition rates.

L. 198. Related to spontaneous rate, is there any relationship between spont rate and type of neuron, sync+ vs. sync-? STD has been hypothesized to be more prominent in neurons with low spont rates (thus not in an adapted state prior to activation).

In response to the reviewer's comments, we compared the spontaneous rates between Sync+ and Sync- neurons. The difference was non-significant (Wilcoxon rank sum test P = 0.68).

LL. 217-239/Fig. 10. This section is quite interesting in relating rate coding properties to fine-scale temporal dynamics. While the sync+/- responses roughly parallel each other in the real vs. simulated data, there are substantial differences. E.g., in the simulation, there is a slow rise time of the sync+ response and complete absence of a sustained response for sync-. Conversely, there is likely to be substantial variability within the actually neural data. Are there parameters in the model that can be varied to produce the variability observed in the real neurons?

The absence of sustained responses in Sync- seems to be due to noise and variation of how much the neurons adapt to a given repetition rate. Adaptation rates ($\mathbf{A_D}$) across stimulus repetition rates vary strongly among Sync- neurons (See S8 Fig.). The sustained response therefore represents the average response.

For Sync+ responses, this model does not take into account adaptation from upstream areas of the auditory pathway. For high frequency stimuli such as pure tones, adaptation can happen even before reaching the auditory cortex (Sumner and Palmer 2012, Malmierca et al 2009) Therefore, the amplitude of excitatory inputs to cortical neurons would be smaller than if there was no such adaptation. In this model, excitation is directly proportional with the high rep rate of pure tones, and therefore produce a very strong and ramping response due to the linear summation of excitatory conductances.

L. 226. Should "differ with" be "differ from" ?

We have corrected this in the revised manuscript

LL. 307-316. Are the any observations of synchronization outside of primary auditory cortex? Or are the authors arguing that R vs. A1 provide a hierarchy parallel to S1 and S2?

The functional role of R, RT and Auditory belt areas are still unclear. Neurons in R and RT can synchronize too, although the synchronization limit is typically more sluggish than AI. R and RT share some similarity to S2: in both regions non-synchronized +/- monotonic neurons are

observed (this is not observed in S1, and at much low proportions in A1). So essentially the temporal-to-rate transformation occurs for a similar range of repetition rate (10-45 Hz) between S1->S2 and AI-> R/RT, however auditory cortex still exhibits some degree of temporal fidelity in higher areas. A similar strategy may exist in auditory belt regions, albeit with neurons responsive to more spectrally complex sound, however this is outside the scope of our paper.

LL. 317-324. These caveats seem relevant, but very limited in their focus. Neural networks are quite complicated and it seems like network effects as well as other single cell mechanisms (like Ih currents) could play a role in producing rate responses. It seems like the topic of alternative models deserves a lot more attention in the Disucssion.
This comment has been addressed with the reviewers first major concern.

L. 368. Is code available for the simulations? Or can some more details be provided about how the simulations were executed?
The code is available on the Github repository under the following link: https://github.com/dbendor/EI-model. The github link was provided in the original manuscript

L. 371. Is the choice of 10 E and I inputs important? Or does the N here simply scale inversely with the strength of the synapses to produce the desired behavior?
The choice of 10E and 10I inputs originates from the earlier model that this work is based on (Wehr and Zador 2003, Bendor 2015). Although the strength of synaptic input scales inversely with N, multiple inputs are important for emulating more biologically realistic conditions such as temporal jitter and other similar noise to synaptic input.

Fig. 10c. The colors are a big difficult to distinguish and the asterisks are quite small.
We have modified the figures in the revised manuscript to implement the reviewers comment.

General note on figures: The current color maps do not print well in black and white.

Supplemental figures: Please indicate in legends when simulated vs. real data are used.
We have corrected this in the revised manuscript

Reviewer #3:

I have no issues with the modeling framework presented in the paper - the integrate and fire component of the model has been previously used and validated, and the synaptic depression extension follows standard approaches (due to Tsodyks, Abbott, etc). My main criticism is more to do with the writing, interpretation, and generalizability. Parts of the manuscript are not particularly clear, and the motivation is lacking. The modelling also seems

specifically tailored to stimulus-synchronizing monotonically tuned neurons. It is not clear what fraction of the cortical population this represents, and I was left constantly asking myself about how generalizable the modelling framework is, with respect to the huge diversity of cell-types and responses present in the cortex. In addition, there are twelve figures. Many of these seem somewhat superfluous to the narrative (and are not referred to in the text) and could perhaps be moved to the supplementary information. Others could perhaps be combined.
In response to the reviewer's concerns, we have attempted to rewrite the manuscript and address these issues, in particular the introduction and the discussion, to better clarify the motivation behind this manuscript. In addition, we have addressed the reviewers comment below on Synchronized non-monotonic neurons in the revised manuscript.
We have also removed the multiplexing section in response to the comments of several reviewers, that has helped reduced the number of figures. We have also moved non-essential figure subplots to create new supplementary figures.

Specific Comments

1) Line 50. With regards to the comment about "brain regions downstream from auditory cortex" - It's not clear what you are referring to here - can you give a specific example?
The mid and Lateral PFC in primates or equivalent areas (such as the orbital gyrus in ferrets (bagur et al 2019)) receives input from many sensory areas including auditory areas (STG in humans, A1 in macaques). We have added specific examples and corresponding citations in the revised manuscript.

2) Lines 55-60. I'm a little confused about the motivation here, specifically with the final question about "how could the brain generate these types of neural representations". What "types" are you referring to - are you simply asking how can the brain generate positive/negative monotonic functions? This needs to be made more explicit.
We have now modified the sentence to read:
"How could the brain generate both positive and negative monotonic tuning in response to a given input?"

3) Related to the comment on line 55, about "rate coding taking the form of positive and negative monotonic tuning". What about non-monotonic tuning (also somewhat ubiquitous in cortex) - would you not call this rate coding?
We agree with the reviewer's comment, and have modified the sentence so that it doesn't exclude other types of rate coding. We have also expanded our analysis to include non-monotonic tuning, which is observed in the model (an intermediate levels of adaptation) and in the real data (page 5 of the revised manuscript).

4) Line 139. Figure 4 isn't really talked about in the text. If it's just a passing reference then maybe make it a supplemental figure?
Please see response to question 5

5) Similar to previous comment, but with Figure 5. Panels E and F are the only panels that are explicitly referred to in the text. If these are not crucial to the story, then perhaps consider placing in supplement.
Based on the reviewer's comment we have made Figure 5A-D into a supplementary figure, and combined figure 4 and figure 5E,F into one figure. While we agree with the reviewer that these figures are not studied in detail in the text, both figure 4 and 5 are outputs of the simulations and a visual comparison between real and model neurons (especially raw data examples) we believe to be critical to the reader's interpretation of the model. We have included the following text in page 3 to better clarify what the readers are seeing in these figures:

For our simulated neurons, A_D values were determined (Fig.4a) so that simulated Sync+ and Sync- neurons matched real neurons in both trial-by-trial spiking activity (Fig.4) and average population activity (S5 Fig). Out of 107 synchronized neurons, 26 neurons were classified as Sync+ (Fig.4c) and 27 were classified as Sync- (Fig.4e). Both simulated (Fig.4b, d) and real (Fig.4c, e) neurons showed stimulus-synchronised driven activity to stimuli, with both simulated (Fig.4d) and real Sync- neurons showing a reliable adaptation of firing rate to higher stimulus repetition rates. Monotonicity was significant for both Sync+ (Spearman correlation coefficient ρ = 0.91, P < 0.001) and Sync- (ρ = 0.85, P = 0.012) simulated neurons (Fig.4f,g), and temporal fidelity over the range of repetition rates spanning flutter perception was maintained despite adaptation (Fig.4b-e, Vector Strength (VS)>0.1, and Rayleigh statistic>13.8, P < 0.001).

6) In the model robustness section, it's clear what has been done but not why. Could you add a few sentences at the beginning of the section to address the biological and mathematical relevance and necessity of adding noise in this way? What is actually being achieved?
The aim of this section is to demonstrate that although the manuscript focuses on the output of a specific set of parameters (adaptation, excitatory, inhibitory input amplitude, noise level and spontaneous rate, temporal jitter of input), Sync + and Sync- responses can be obtained using a wide range of these parameters. Neural responses to the same stimulus can be highly variable both between different neurons and within the same neuron between different trials. We therefore show in this section that the model's ability to produce Sync+ and Sync- responses is robust to changes in parameters.
In response to the reviewer's comment, we included the following sentence in the revised manuscript:
Although our simulated neurons show similar Sync+ and Sync- responses to averaged real Sync+ and Sync- responses, we observed that in real neurons, innate properties such as spontaneous rate and onset response to stimuli varied among neurons within the same category. We therefore examined whether the model's ability to produce Sync+ and Sync- simulated neurons were robust to changes in initial conditions of the model.

7) Could Figures 6 and 7 be combined into one figure looking at model robustness?

We’ve considered the reviewer’s suggestion, but decided that combining these two figures lowered the overall readability. We have however removed part of these figures and made them supplementary.

8) The section on “pure tone responses” comes a little out of left field. It’s not made particularly clear why this section is necessary, or why it is related to the rest of the paper.
Our model was fitted to real Sync+ and Sync- neuron responses to repetition rates within flutter. The model however suggests that Sync+ and Sync- responses result from innate bio-physical properties of the neuron, as opposed to a stimulus specific response. If the former is true, simulated Sync+ and Sync- neurons should respond to other simple stimuli in the same way as real Sync+ and Sync- neurons. We also felt that using a different stimulus type, not used to classify the neuron’s response, provides a more powerful prediction. We studied the model’s response to pure tones, simple stimuli known to show robust responses in Primary auditory cortex, to test this hypothesis.

9) The demultiplexing section is an interesting addition, but it doesn’t seem clear that this idea is really biologically relevant. In practice, a sound is made up of *lots* of acoustic features (of which, things like frequency, level, repetition rate) are only a few. Is there *any* reason to believe, or any evidence to suggest, that every potential acoustic feature has to have monotonic tuning?
In response to comments of multiple reviewers, we’ve decided to remove this section, and will focus a separate manuscript on the concept of multiplexing.
10) Can the authors provide additional simulations that suggest that summing or subtracting firing rates can lead to demultiplexing of more than two acoustic features?
Please see response to 9

11) What fraction of the data set are the Sync+ and Sync- neurons (i.e how many non-monotonic neurons are there)? If this model is only suitable for a sub-set of cortical neurons (the monotonic ones), then the implications of biological differences in the circuitry underlying non-monotonic neurons needs to be discussed.
Please see response to 12
12) Similarly, the neuron’s studied seem to be only those that synchronize to the stimulus. What fraction are these?
As the methods show, 107/274 were Sync neurons Out of these neurons, 53 were Sync+ or Sync- and 54 neurons showed synchronised non-monotonic responses.
Based on the reviewer’s comment we have also expanded our analysis to address sync NM neurons. Sync NM neurons are generated with intermediate values of synaptic depression, and our analysis of real Sync NM neurons also show intermediate levels of adaptation. This suggests that the strength of synaptic depression varies in real neurons along a continuum, and a consequence of this is monotonic tuning to repetition rate at the two extremes of this continuum (and non-monotonic tuning in the middle).

13) Could Figures 11 and 12 be combined into one figure?
Please see response to 9

Minor Points.
1) Line 11: missing article? "represented temporally by the phase-locked..."
2) Line 29: extra s? "mechanisms that generate"
3) Line 31: pluralize code? "generate monotonic rate code"
4) Line 193: extra s in "neurons populations".

We thank the reviewer for pointing out these errors. Corrections for points 1-4 have been made in the revised manuscript.
