# Supplementary material for: The role of adaptation in generating monotonic rate codes in auditory cortex
Source: PLoS Comput Biol. 2020 Feb 18;16(2):e1007627. doi: 10.1371/journal.pcbi.1007627 (PMC7048304; doi:10.1371/journal.pcbi.1007627)
Supplement: Attachment — Submitted filename: response to reviewers review2.docx [file pcbi.1007627.s013.docx]

We thank the reviewers for their constructive and helpful remarks and greatly appreciate the efforts they made in providing detailed comments on our manuscript. The following is a point-by-point list of all changes made in response to the comments by the three referees. All page and figure numbers referred to here are those of the revised manuscript.

Reviewer #1:

**1. The title of Figure 7, I think, is misleading. I HIGHLIGHT the problematic parts: "Adaptation between INDIVIDUAL synaptic inputs for REAL neurons and different models". I don't think that panel B, which deals with the real neurons, has anything to do with individual synaptic inputs. It shows the strength of adaptation measured as a ratio between the 1st and 2nd or 3rd pulses. The connection to individual synaptic inputs is a conjecture here, as their adaptation was not measured but inferred. Please replace the title with one that would accurately describe what the figure actually shows.**

In response to the reviewer’s comments, we have modified the title to “adaptation between individual **acoustic events”,** as this would more accurately represent what is being studied in the figure.

**2. The new paragraph starting on line 329 is poorly written. Please check the grammar throughout, starting from line 329.**

We have modified the paragraph in line 329 with the following:

*Second, our model does not account for different sources of inhibition – such as top-down inhibitory feedback or cortical inhibition from different interneurons - that could affect excitatory pyramidal cells in the auditory cortex. In particular, there has been increasing evidence of top-down signals mediating rapid, behaviourally driven modulatory effects in the auditory cortex [46, 61-63]. A recent study has shown that a network of Parvalbumin-positive (PV) inhibitory neurons may mediate such top-down control by rapidly and efficiently modify sensory responses in auditory cortex in mice [63]. However, the timescale of adaptation occurring in Sync+ and Sync- neurons was within the order of milliseconds, and therefore the contribution of such top-down inhibition seemed unlikely. In addition, the spontaneous rate of cortical neurons did not decrease for stronger adaptation as it did with activation of PV inhibitory neurons in the mentioned study. Nonetheless, we cannot rule out possible effects of inhibition from other interneurons with shorter, more transient timescales as observed in somatostatin (SOMs) and vasoactive intestinal polypeptide-expressing (VIPs) interneurons [64,65]. Further studies using optogenetics to manipulate different types of inhibitory neurons may provide additional insight on how inhibition affects adaptation in the auditory cortex.*
